# Supplementary material for: Interfacial Charge Transfer and Ultrafast Photonics Application of 2D Graphene/InSe Heterostructure
Source: Nanomaterials (Basel). 2022 Dec 28;13(1):147. doi: 10.3390/nano13010147 (PMC9824543; doi:10.3390/nano13010147)
Supplement: Supplementary file 1 [file nanomaterials-13-00147-s001.zip › nanomaterials-2119453-supplementary.pdf]

nanomaterials

MDPI

*Supporting Information*

# Interfacial Charge Transfer and Ultrafast Photonics Application of 2D Graphene/InSe Heterostructure

**Jialin Li [1], Lizhen Wang [2], Yuzhong Chen [2], Yujie Li [2], Haiming Zhu [2], Linjun Li [1,3,*] and Limin Tong [1,3]**

1 State Key Laboratory of Modern Optical Instrumentation, College of Optical Science and Engineering, Zhejiang University, Hangzhou 310027, China
2 Center for Chemistry of High-Performance & Novel Materials, Department of Chemistry, Zhejiang University, Hangzhou 310027, China
3 Intelligent Optics & Photonics Research Center, Jiaxing Research Institute, Zhejiang University, Jiaxing 314000, China
* Correspondence: lilinjun@zju.edu.cn

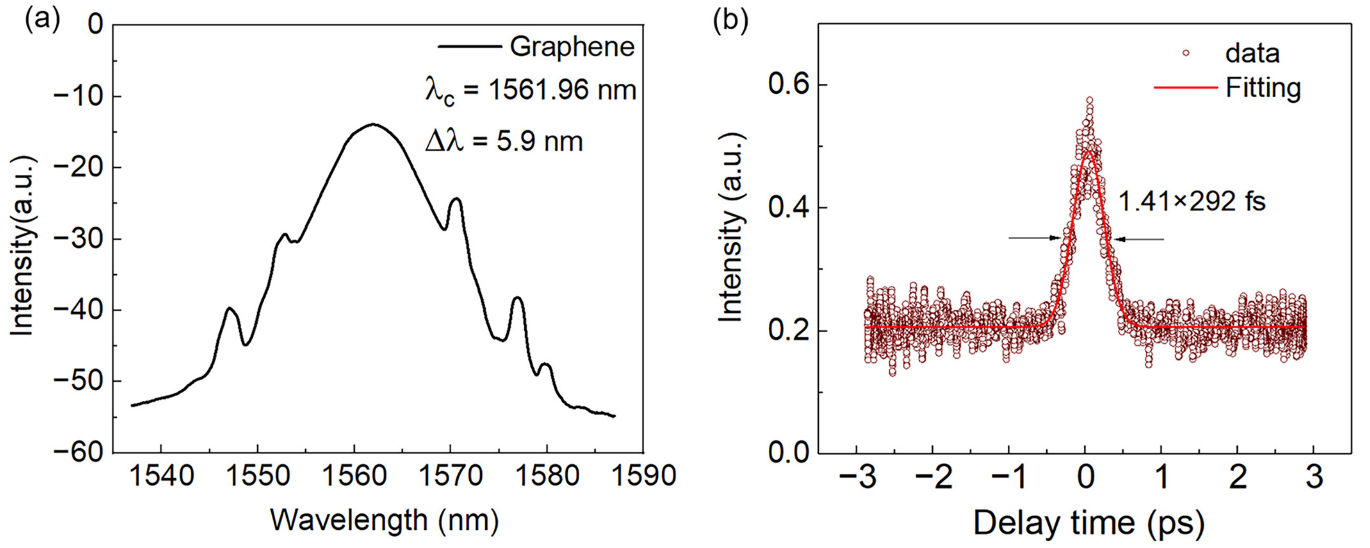


**Figure S1.** Mode-locked laser pulse based on only graphene (before stacked with InSe) SA. (a) Optical spectrum. (b) AC trace.

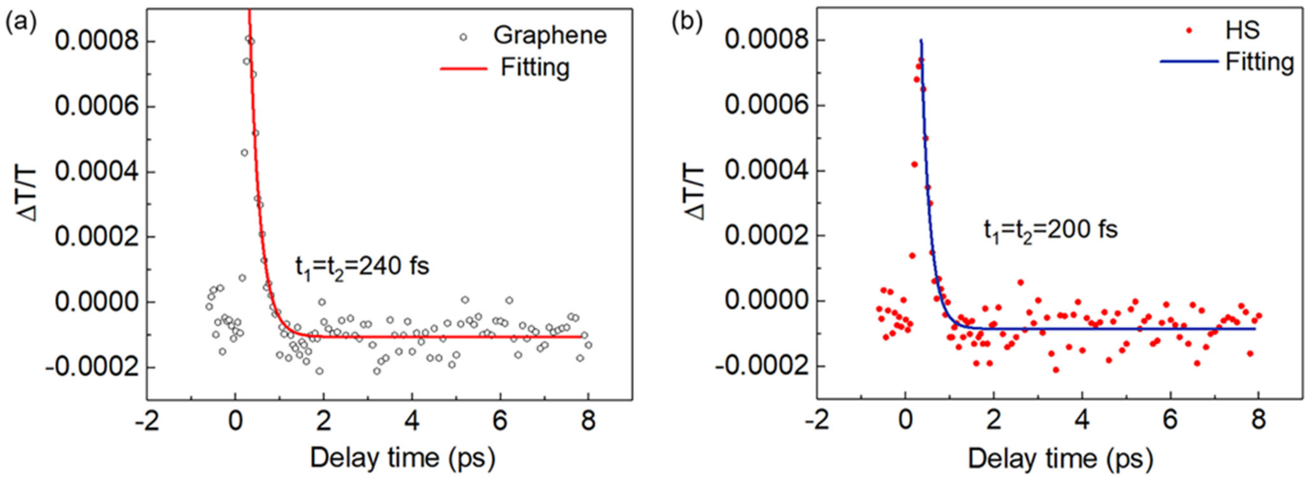


**Figure S2.** The TA signal of (a) graphene and (b) G/InSe HS with pump and probe wavelengths of 1350 nm and 1566 nm respectively.

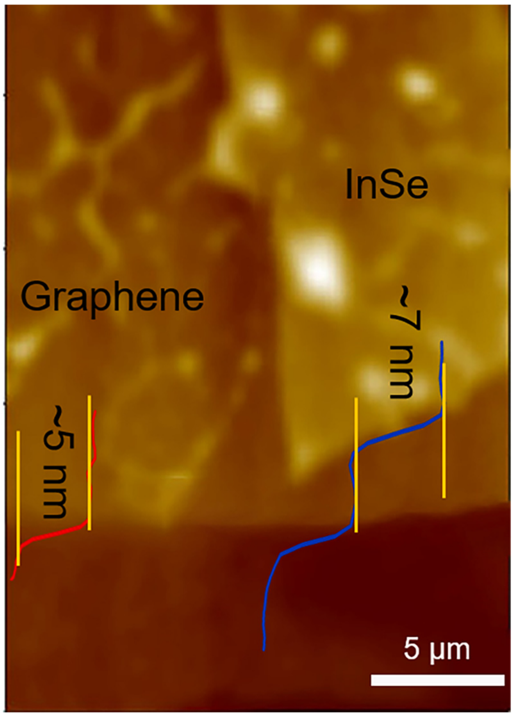


**Figure S3.** AFM image of graphene and InSe.
